# The Effects of Cr and Mo Additions on the Corrosion Behavior of Fe–Al Alloys in 0.5 M H_2_SO_4_ and 3.5 wt.% NaCl Aerated Aqueous Solutions

**DOI:** 10.3390/ma19010190

**Published:** 2026-01-04

**Authors:** Chao-Chun Yen, Ting-Hsu Chang, Yun-Xian Lin, Meng-Ying Wu, Shiow-Kang Yen

**Affiliations:** Department of Materials Science and Engineering, National Chung Hsing University, Taichung 40227, Taiwan; murdocdepp@gmail.com (C.-C.Y.); shes96293@gmail.com (T.-H.C.); yxlin0210@gmail.com (Y.-X.L.); slimu16882013@gmail.com (M.-Y.W.)

**Keywords:** Fe-Al alloy, potentiodynamic polarization, passive film, ICP-MS

## Abstract

This study aims to investigate the effects of Cr and Mo added to Fe-Al alloys on their corrosion behavior in acidic and chloride-containing environments. Corrosion tests were carried out in 0.5 M H_2_SO_4_ and 3.5 wt.% NaCl aerated aqueous solutions. X-ray diffraction analyses reveal that all alloys exhibited predominantly body-centered cubic structures in the homogenized states. In the 0.5 M H_2_SO_4_ solution, the addition of Cr can effectively reduce the critical current density; however, the anodic and cathodic polarization curves still intersected three times, similar to the alloy without the addition of Cr, resulting in three corrosion potentials. With the further addition of Mo, the critical current density became much lower, leading to a single corrosion potential. In the 3.5 wt.% NaCl solution, the addition of Cr alone markedly improved the pitting resistance of Fe-Al alloys, while the further addition of Mo broadened the passive region and increased the pitting potential. The analysis of ion concentrations was consistent with the potentiodynamic polarization results, verifying the stabilization of Mo on the passive film. It is evident that the addition of Cr promotes passivation of the Fe-Al alloy, and the further incorporation of Mo enhances this effect even more significantly. The related corrosion mechanisms are discussed with Nerst equations of metal–metal oxides and their solubility products (K_sp_).

## 1. Introduction

In today’s era of advanced technology, iron and aluminum are ubiquitous in various aspects of daily life, transportation, and construction. Compared to other metals, they are relatively inexpensive and readily available [1,2,3]. Research on Fe-Al-based alloys has been conducted since the previous century [4,5,6]. Iron–aluminum alloys possess several attractive properties, including low density, high strength, and excellent resistance to high-temperature oxidation [7,8]. Aluminum is lighter than iron and has a larger atomic radius. These two characteristics contribute to the weight reduction of Fe-Al alloys, decreasing the density from 7.87 g/cm^3^ for pure iron to 5.59 g/cm^3^ for Fe-50Al. Therefore, Fe-Al alloys are considered lightweight materials in comparison to traditional stainless steels and nickel-based superalloys [9].

At elevated temperatures, aluminum readily reacts with oxygen to form an oxide layer. When the aluminum content is sufficient, a dense and stable layer of Al_2_O_3_ forms on the surface, which effectively prevents further oxidation of the metal. For this reason, aluminum is commonly added to various alloys to enhance their high-temperature oxidation resistance [10,11]. The passive film formed on binary iron–aluminum alloys provides good corrosion resistance in neutral aqueous solutions that do not contain chloride ions. However, when chloride ions are present in the solution, localized corrosion can occur due to the penetration of chloride ions, significantly reducing the corrosion resistance of the binary Fe–Al alloys [12]. Therefore, improving corrosion resistance remains a critical issue and a major challenge for the development of Fe–Al alloys.

To improve the corrosion resistance of alloys in various environments, scientists commonly incorporate additional elements into the alloy matrix, such as chromium [12,13], niobium [14], zirconium [14], titanium [15,16], and molybdenum [17,18,19,20,21]. The addition of chromium to Fe–Al alloys is widely recognized as an effective strategy for enhancing their corrosion resistance [12]. Chromium can form a Cr_2_O_3_ layer on the surface, which stabilizes the passive film and significantly improves the corrosion resistance of alloys. Furthermore, studies have shown that chromium not only enhances corrosion resistance but also improves the room-temperature ductility of Fe–Al alloys [13]. M. Negache and colleagues studied the effects of adding chromium, niobium, and zirconium to Fe–Al alloys. Their results showed that chromium alone significantly enhanced corrosion resistance, while the combined addition of chromium and niobium led to even greater improvements in corrosion performance [14]. Wann-Chiun Luu et al. found that the addition of titanium to Fe–Al alloys resulted in the formation of a thicker and more stable passive film, leading to superior corrosion resistance in acidic, alkaline, and neutral environments [15]. Molybdenum is another element known to significantly enhance corrosion resistance in various alloys [17,18,19,20,21]. During passive film formation, Mo is oxidized into MoO_2_, MoO_3_, and MoO_4_^2−^. These species contribute to strengthening the passive film through chemical adsorption and bonding with the oxide layer [17,18]. In addition, molybdenum can act as a sacrificial anode element, preferentially reacting to protect other metals from corrosion [19]. In alloys containing both Mo and Cr, a bilayer structure composed of MoO_4_^2−^ and Cr_2_O_3_ may form, which improves resistance against chloride ion attack [20]. However, excessive molybdenum content may lead to the precipitation of secondary phases along grain boundaries, resulting in intergranular corrosion and a reduction in overall corrosion resistance. In the study conducted by Jianming Wang et al., AlCrFeNi_3_Mo_x_ (x = 0, 0.1, 0.2, 0.3, 0.4) alloys exhibited optimal corrosion resistance at Mo = 0.3. When Mo = 0.4, the formation of excessive σ-phase at grain boundaries created corrosion-sensitive regions, thereby decreasing the corrosion resistance [21].

Ferritic stainless steel is a type of alloy steel characterized predominantly by a body-centered cubic (BCC) structure, such as AISI 430 stainless steel. It primarily relies on chromium as the corrosion-resistant element and contains little to no nickel. Due to the absence of nickel, ferritic stainless steels are generally more cost-effective and price-stable compared to austenitic stainless steels, which typically contain 8 to 10 wt.% nickel [22]. In terms of corrosion resistance, although the chromium content in ferritic stainless steel is sufficient to form a protective chromium oxide layer, the absence of stabilizing elements such as nickel and molybdenum makes the passive film less resilient. As a result, the self-healing ability of the passive film is limited once it is damaged, reducing the alloy’s ability to suppress further corrosion. In marine environments, where electrochemical conditions can vary significantly, the dissolution of active sites in ferritic stainless steel is accelerated, leading to the aggravation of localized corrosion. Consequently, the application of ferritic stainless steels in high-chloride seawater environments is considerably restricted [23].

Both Fe–Al alloys and ferritic stainless steels are free of nickel in their compositions, which gives them a notable advantage in terms of raw material cost compared to nickel-containing alloys. Moreover, due to the presence of aluminum, Fe–Al alloys possess lower density and overall weight than ferritic stainless steels. These advantages in both cost and weight endow Fe–Al alloys with considerable potential for broader application and further development. This study aims to investigate the effects of Cr and Mo added to Fe-Al alloys on their corrosion behaviors in acidic and chloride-containing environments, respectively, and the Nernst equation will be employed to calculate the redox equilibrium potentials (E) of metal oxides/hydroxides proceeding through the electrochemical reactions in dynamic polarization tests and the solubility products (K_sp_) of their hydroxides can be corresponding to the stability of passivation film, as reported in previous studies [24].

## 2. Materials and Methods

The alloys were prepared using high-purity metals (99.99 wt.%), including Al, Cr, Fe, and Mo, combined in specific proportions, as listed in Table 1. The metals were placed in a water-cooled copper crucible within a vacuum arc melting furnace and melted under an argon atmosphere. The melting process was repeated three times to ensure chemical homogeneity. As-cast samples were then sealed in evacuated quartz tubes and subjected to homogenization heat treatment at 1200 °C for 48 h. After homogenization, the samples were furnace-cooled to 800 °C and subsequently water-quenched to room temperature to obtain homogenized alloy specimens.

Both as-cast and homogenized specimens were machined into dimensions of 5 mm × 5 mm × 2 mm using wire electrical discharge machining (EDM). To ensure electrical conductivity, a copper wire was spot-welded to the back of each specimen, and the samples were cold-mounted in epoxy resin. Prior to potentiodynamic polarization testing, the exposed surface area (0.25 cm^2^) was sequentially ground using silicon carbide (SiC) papers ranging from grit #100 to #2500, followed by polishing with 3 µm diamond suspension and final polishing with 0.02 µm colloidal silica. The polished surfaces were then ultrasonically cleaned in deionized (DI) water for 15 min to remove residual polishing agents and surface contaminants.

Phase identification was performed using a X-ray diffractometer (XRD) (BRUKER, model D8 Controller, Billerica, MA, USA) with Cu Kα radiation (wavelength = 1.542 Å). The measurements were conducted under operating conditions of 40 kV and 25 mA, with a scanning speed of 0.005° per second over a 2θ range from 20° to 100°.

Hardness measurements were conducted at seven locations (*n* = 7) on each specimen, with a minimum spacing of 3 mm between points, to obtain an average value. A Vickers hardness tester (Shimadzu, model HMV-G21, Japan) was used for testing, applying a load of 19.61 N with a dwell time of 15 s.

Potentiodynamic polarization tests were conducted three times on each specimen using a conventional three-electrode system, consisting of a reference electrode, a counter electrode, and a working electrode. A commercial Ag/AgCl electrode in saturated KCl solution served as the reference electrode, while a platinum electrode was used as the counter electrode. The working electrode was a polished specimen with an exposed area of 0.25 cm^2^. The tests were performed at room temperature in aerated 0.5 M H_2_SO_4_ and 3.5 wt.% NaCl aqueous solutions. The potential of the working electrode was swept from –1.2 V (versus open circuit potential, vs. E_corr_) to 1.2 V (vs. E_ref_), at a scan rate of 0.167 mV/s. Polarization measurements were carried out using a Multi-Channel Potentiostat VSP system operated with the accompanying EC-Lab software (Bio-Logic, Paris, France). Prior to each test, the specimen was immersed in the electrolyte for 30 min to stabilize the surface condition.

The surface morphologies of the specimens before and after potentiodynamic polarization testing were examined under high-vacuum conditions (10^−6^ torr) using a scanning electron microscope (SEM) (JEOL Ltd., JEOL IT-100, Tokyo, Japan) equipped with secondary electron imaging (SEI), backscattered electron imaging (BEI), and energy-dispersive X-ray spectroscopy (EDS) for surface morphology observation and elemental analysis.

During the polarization tests conducted in aerated 0.5 M H_2_SO_4_ and 3.5 wt.% NaCl solution, the concentration of metal ions released from Fe_80_Al_20_ and Fe_60−x_Al_20_Cr_20_Mo_x_ (x = 0, 2.3, 4.7) alloys was analyzed using inductively coupled plasma mass spectrometry (ICP-MS). For quantitative analysis, approximately 15 mL of the electrolyte solution was collected from the central region between the working and counter electrodes at applied potentials of E_corr_, 0.2 V, 0.6 V, 0.86 V, 1.0 V, and 1.2 V for subsequent measurement.

## 3. Results and Discussion

### 3.1. Material Properties

#### 3.1.1. Phase Structure Analysis of Alloys

**Figure 1:**
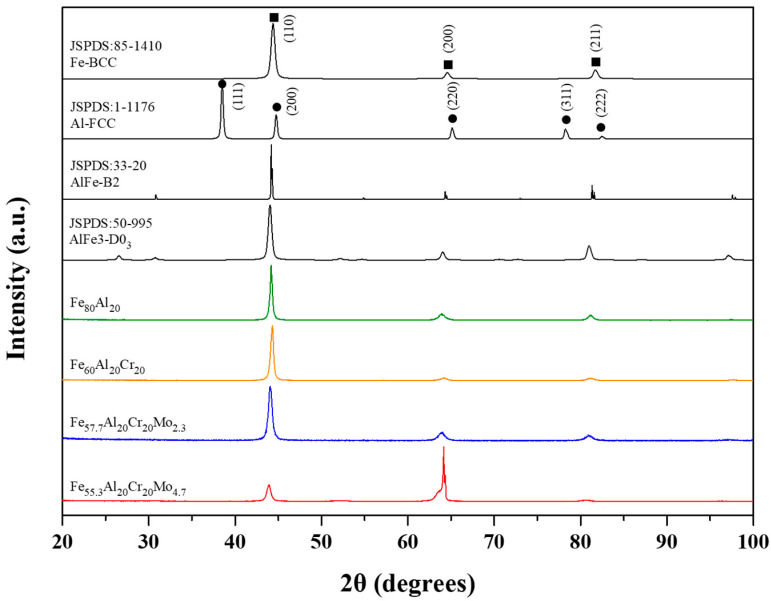
The XRD patterns of homogenized Fe_80_Al_20_, Fe_60_Al_20_Cr_20_, Fe_57.7_Al_20_Cr_20_Mo_2.3_, and Fe_55.3_Al_20_Cr_20_Mo_4.7_ alloys.

The X-ray diffraction (XRD) analysis results of Fe_80_Al_20_, Fe_60_Al_20_Cr_20_, Fe_57.7_Al_20_Cr_20_Mo_2.3_, and Fe_55.3_Al_20_Cr_20_Mo_4.7_ alloys after homogenization are shown in Figure 1. All Fe-Al-based alloys exhibit three major diffraction peaks corresponding to the disordered A2 structure, indicating that their crystal structures remain in the body-centered cubic (BCC) phase. After homogenization at 1200 °C, no characteristic peaks of the ordered B2 or D0_3_ structures were obviously detected, suggesting that high-temperature treatment did not induce a noticeable ordering transformation and that the alloys remained predominantly in the disordered A2 phase.

#### 3.1.2. Hardness Test Results of the Alloys

Table 2 presents the hardness test results of the Fe_80_Al_20_, Fe_60_Al_20_Cr_20_, Fe_57.7_Al_20_Cr_20_Mo_2.3_, and Fe_55.3_Al_20_Cr_20_Mo_4.7_ alloys. The results indicate that the addition of Cr alone to the Fe-Al alloy did not lead to a significant improvement in hardness. However, when both Cr and Mo were introduced, the alloys exhibited a remarkable increase in hardness, which further improved with increasing Mo content, indicating that the strengthening effect of Mo is greater than that of Cr. According to the study by C. G. McKamey et al., the addition of an appropriate amount of Mo to Fe-Al alloys helps to increase the recrystallization temperature and suppress grain growth, thereby achieving the effect of grain refinement strengthening. In addition, Mo also contributes positively to solid-solution strengthening and precipitation hardening [25].

### 3.2. Corrosion Behavior Analysis in 0.5 M H_2_SO_4_ Aerated Aqueous Solution

#### 3.2.1. Electrochemical and Potentiodynamic Polarization Analysis of the Alloys in H_2_SO_4_ Aerated Aqueous Solution

This study employs the Nernst equation to calculate the redox equilibrium potentials (E) of metal oxides/hydroxides proceeding through the electrochemical reactions as summarized in Table 3 at different pH values. The redox equilibrium potentials of corresponding reactions are expressed in Equation (1) or Equation (2):

E = m OH^−^/ n e^−^ × (14 − pH) × 0.0591 + E^0^ − 0.197 V (1)

E = −m H^+^/ n e^−^ × pH × 0.0591 + E^0^ − 0.197 V (2)

In these equations, *m* denotes the number of moles of OH^−^ or H^+^, *n* represents the number of moles of electrons (e^−^) involved in the redox process, *E^0^* is the standard redox equilibrium potential [26,27], and 0.197 V corresponds to the potential difference between the Ag/AgCl reference electrode used in this study and the standard hydrogen electrode (SHE).

By incorporating the solubility product constants (K_sp_) of metal oxides and hydroxides, the possible composition of passive films formed during potentiodynamic polarization tests can be effectively predicted. The K_sp_ values of various metal oxides/hydroxides are summarized in Table 4 [28,29], and these values play a critical role in evaluating the corrosion resistance of alloys [24,30]. Within electrochemical corrosion mechanisms, K_sp_ provides a basis for determining the formation and stability of oxides or hydroxides, thereby enabling the assessment of their passivation capability. In general, a lower K_sp_ indicates reduced solubility, lower ionic release, and a greater tendency to form a stable and compact passive film on the metal surface, which effectively blocks corrosive media and suppresses further metal dissolution. Conversely, a higher K_sp_ corresponds to higher solubility, hindering the formation of a durable protective layer and resulting in diminished passivation performance. Therefore, K_sp_ serves as a key parameter for predicting passive film stability and assessing the corrosion resistance behavior of alloys.

**Figure 2:**
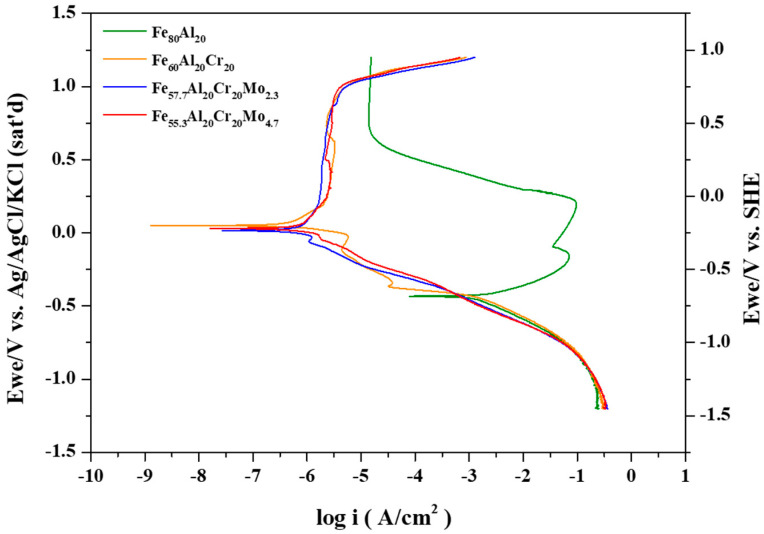
Representative potentiodynamic curves of Fe_80_Al_20_, Fe_60_Al_20_Cr_20_, Fe_57.7_Al_20_Cr_20_Mo_2.3_, and Fe_55.3_Al_20_Cr_20_Mo_4.7_ in 0.5M H_2_SO_4_ aerated aqueous solutions.

The representative potentiodynamic polarization curves of the Fe_80_Al_20_, Fe_60_Al_20_Cr_20_, Fe_57.7_Al_20_Cr_20_Mo_2.3_, and Fe_55.3_Al_20_Cr_20_Mo_4.7_ alloys in oxygenated 0.5 M H_2_SO_4_ solution are presented in Figure 2. The corresponding electrochemical parameters, including the open-circuit potential after 30 min of immersion (E_open_), corrosion potential (E_corr_), corrosion current density (i_corr_), passive current density (i_pass_), passivation potential (E_trans_), and critical current density (i_crit_), are summarized in Table 5 The polarization behavior of these alloys can be rationalized by considering the redox potentials (E) of the constituent metallic elements and the solubility products (K_sp_) of their hydroxides, as reported in previous studies [24,30].

For the Fe_80_Al_20_ alloy, the standard electrode potential (E^0^) of the electrochemical reaction from Al to Al^3+^ is −1.859 V (Ag/AgCl); at higher potentials, aluminum preferentially forms Al_2_O_3_ and Al(OH)_3_, as listed in Table 3 (Reactions 3 and 4). Although Al(OH)_3_ exhibits a relatively low solubility product constant (K_sp_ = 3 × 10^−34^), in sulfuric acid aqueous solution at pH = 0.47, a substantial amount of Al ions (approaching 10^6^ as calculated from K_sp_ and pH) remains dissolved in the solution. On the other hand, the E^0^ of the electrochemical reaction from Fe to Fe^2+^ is −0.64 V; therefore, iron is more susceptible to preferential dissolution into the electrolyte during the initial stage of corrosion, due to the relatively high solubility product constant of (K_sp_ = 2 × 10^−15^) Fe(OH)_2_, which becomes dominant at the potential increasing to −0.064 V by Reaction (8), finally leading to the greater i_crit_ of 70,300 μA/cm^2^ and i_pass_ of 64,294 μA/cm^2^. When the potential is further increased to 0.043 V, Reaction (10) becomes the prevailing reaction pathway; in other words, Fe(OH)_2_ is further oxidized to Fe(OH)_3_. Owing to the extremely low solubility product constant of Fe(OH)_3_ (K_sp_ = 6 × 10^−38^), Fe(OH)_3_ progressively precipitates on the alloy surface, promoting the formation of a denser and more protective passive film. The establishment of this passive film effectively suppresses further metal dissolution, resulting in a substantial reduction in the i_pass_ reduced from 64,294 to 14.5 μA/cm^2^ and ultimately leading to the development of a stable passive state.

In the Fe_60_Al_20_Cr_20_ alloy, the addition of Cr promotes the formation of Cr(OH)_3_ at approximately −0.697 V, much lower than −0.064 V in Reaction (8), as described by Reaction (5) in Table 3. Given the lower K_sp_ (7 × 10^−31^) of Cr(OH)_3_ than that (2 × 10^−15^) of Fe(OH)_2_, Cr(OH)_3_ readily forms and remains stable on the alloy surface, contributing to the formation of a compact and protective passive film, leading to a reduced i_cirt_ from 70,300 to 12,080 μA/cm^2^ and reduced i_pass_ from 14.5 to 2.43 μA/cm^2^. When Mo is added to the alloy, such as Fe_57.7_Al_20_Cr_20_Mo_2.3_ and Fe_55.3_Al_20_Cr_20_Mo_4.7_, it is sequentially oxidized at −0.377 V and 0.355 V to form MoO_2_ and MoO_3_, respectively. These oxides, MoO_2_ and MoO_3_, further reinforce the structure of the Cr-rich passive film, rendering the existing Cr(OH)_3_ layer more compact and robust, finally exhibiting a much reduced i_cirt_ from 12,080 to 78.8 μA/cm^2^. These alloys maintain stable passive films and demonstrate excellent corrosion resistance at potentials up to 1.0 V.

**Figure 3:**
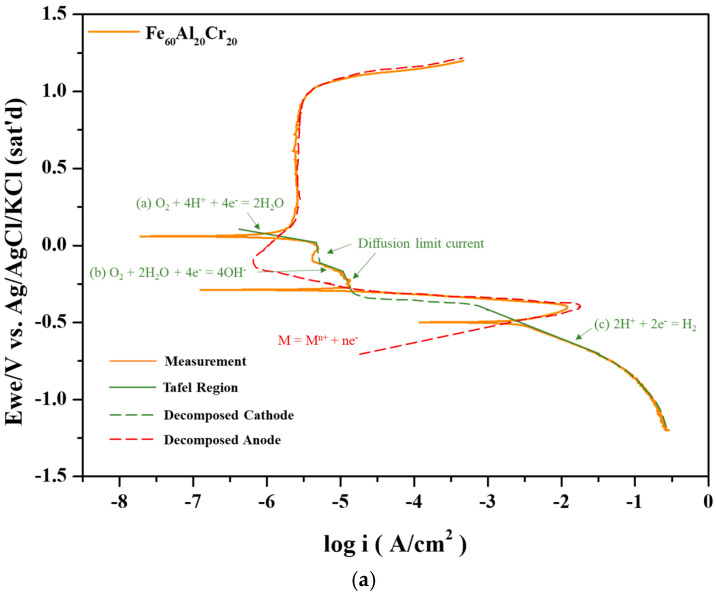

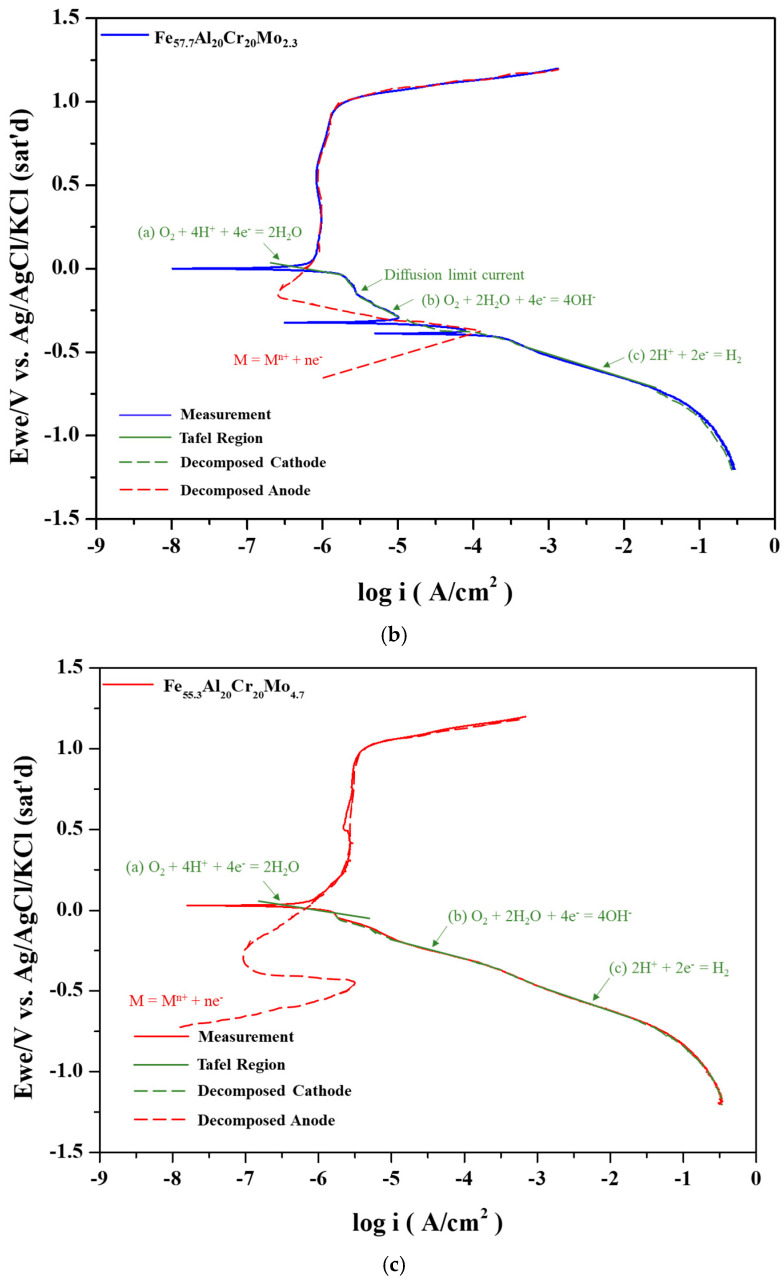
The anodic curves and cathodic curves decomposed from Figure 2 for (**a**) Fe_60_Al_20_Cr_20_, (**b**) Fe_57.7_Al_20_Cr_20_Mo_2.3_, and (**c**) Fe_55.3_Al_20_Cr_20_Mo_4.7_ alloys.

In the Fe–Al system, the polarization curve exhibits only a single corrosion potential since both the critical current density and passive current density are relatively high. However, upon the addition of Cr, the critical current density decreases significantly, and the passivation occurs rapidly, resulting in the intersection with the cathodic polarization curve of O_2_ diffusion limit current density in Reaction (b) and revealing the second corrosion potential. Finally, the anodic passivation curve intersects with the cathodic Reaction (a) at the third corrosion potential, as shown in Figure 3a. Under these conditions, the anodic polarization curve intersects the cathodic polarization curve three times, corresponding to three corrosion potentials. With the addition of a small amount of Mo, the critical current density further decreases, yet the anodic and cathodic curves still produce three intersections, as shown in Figure 3b, similar to Figure 3a. The first corrosion potential lies between the Tafel regions of Reactions (b) and (c); the second corrosion potential is located by the intersection of the anodic curve with the Tafel region of Reaction (b); and the third corrosion potential arises from the intersection between the passive current region and the Tafel region of Reaction (a).

For the Fe_55.3_Al_20_Cr_20_Mo_4.7_ alloy shown in Figure 3c, an even lower critical current density is observed. In this case, the anodic polarization curve no longer intersects Reactions (b) or (c) at a lower potential and instead intersects only with the Tafel region of Reaction (a) at a higher potential, resulting in a single corrosion potential. The three corrosion potentials were also described in a previous report of 304 stainless steel and CoCrFeNi high entropy alloys [24].

#### 3.2.2. Surface Morphologies Before and After Potentiodynamic Polarization Tests in Aerated 0.5 M H_2_SO_4_ Solution

**Figure 4:**
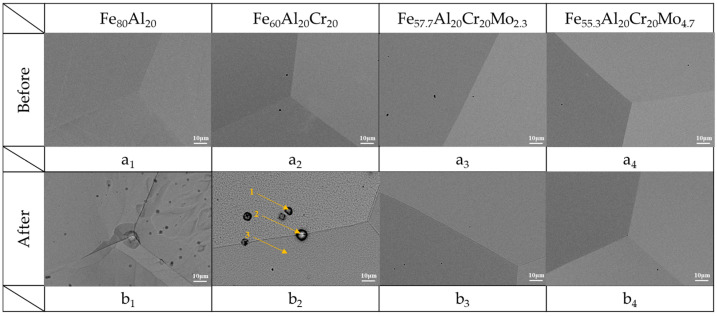
(**a_1_**–**a_4_**) The SEM images of Fe_80_Al_20_, Fe_60_Cr_20_Al_20_, Fe_57.7_Al_20_Cr_20_Mo_2.3_, and Fe_55.3_Al_20_Cr_20_Mo_4.7_ alloys before corrosion testing; (**b_1_**–**b_4_**) the SEM images of the same alloys after exposure to 0.5 M H_2_SO_4_ solution. The accelerating voltage is 20 kV, and the magnification is 1000×.

As shown in Figure 4, the Fe_80_Al_20_ alloy exhibits pronounced surface corrosion, with particularly severe attack occurring along the grain boundaries, accompanied by numerous corrosion spots across the surface. In the Fe_60_Al_20_Cr_20_ alloy, extensive pitting corrosion is observed after testing. According to the EDS analysis listed in Table 6, the locations of these pits correspond to regions that originally contained nitrogen-, oxygen-, and aluminum-bearing compounds. The nitrogen signal originates from AlN. During the melting process, oxygen was largely consumed through surface oxidation reactions, allowing the remaining nitrogen to diffuse into the metal and react with aluminum to form AlN. The formation energy of AlN (−3.46 eV) may be significantly greater than that of FeN, CrN, and MoN, making it the most thermodynamically stable nitride in the system. During corrosion, these compounds dissolve into the solution, resulting in the formation of pits. Consequently, no nitrogen signal is detected at the center of the pits, while nitrogen is detected at the pit edges, such as at point 2 in the figure, corresponding to regions where corrosion products accumulate. These findings demonstrate that the addition of Cr alone is insufficient to suppress the severe corrosion of Fe-Al alloys in 0.5 M H_2_SO_4_ solution.

In contrast, the Mo-containing alloys exhibited only slight intergranular corrosion for Fe_57.7_Al_20_Cr_20_Mo_2.3_, with the overall surface remaining intact and free from noticeable corrosion damage. As the Mo content increased, not only was the excellent corrosion resistance maintained, but the extent of intergranular attack was further reduced. This result confirms that the addition of Mo effectively enhances the intergranular corrosion resistance of the alloys in sulfuric acid environments, which is consistent with the further reduced critical current density observed in the polarization curves shown in Figure 2.

### 3.3. Corrosion Behavior Analysis in 3.5 wt.% NaCl Aerated Aqueous Solution

#### 3.3.1. Electrochemical and Potentiodynamic Polarization Analysis of the Alloys in 3.5 wt.% NaCl Aerated Aqueous Solution

The formation of various metal oxides and hydroxides during the corrosion process occurs through the electrochemical reactions listed in Table 7, and their redox equilibrium potentials (E) can be calculated using the Nernst equations presented in Equations (1) and (2).

Figure 3 presents the potentiodynamic polarization curves of Fe_80_Al_20_, Fe_60_Al_20_Cr_20_, Fe_57.7_Al_20_Cr_20_Mo_2.3_, and Fe_55.3_Al_20_Cr_20_Mo_4.7_ alloys in aerated 3.5 wt.% NaCl solution. The corresponding electrochemical parameters, including the open-circuit potential after 30 min of immersion (E_open_), corrosion potential (E_corr_), corrosion current density (i_corr_), passive current density (i_pass_), and critical current density (i_crit_), are summarized in Table 8. The polarization behavior of these alloys can be rationalized based on the redox equilibrium potentials (E) of the constituent metals and the solubility product constants (K_sp_) of their hydroxides, which together provide a mechanistic understanding of corrosion behavior in aqueous environments, consistent with earlier studies [30].

**Figure 5:**
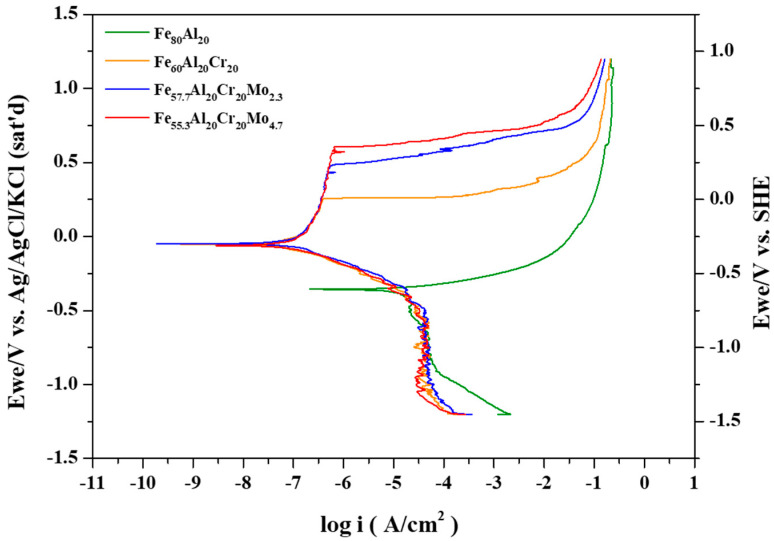
Potentiodynamic curves of Fe_80_Al_20_, Fe_60_Al_20_Cr_20_, Fe_57.7_Al_20_Cr_20_Mo_2.3_, and Fe_55.3_Al_20_Cr_20_Mo_4.7_ in 3.5 wt.% NaCl aerated aqueous solutions.

As shown by the polarization curve of the Fe_80_Al_20_ alloy in Figure 5, the alloy fails to form an effective protective passive film during the potentiodynamic polarization test. According to Reactions (13) and (14), aluminum can form Al(OH)_3_ and Al_2_O_3_ at low potentials, which deposit on the specimen surface. In addition, based on Reaction (19), Fe is converted to Fe(OH)_2_ with the formation of Fe^2+^ when E_corr_ −0.356 V is higher than −0.425 V. However, Fe^2+^ can further transform into Fe(OH)_3_ at lower potentials by Reaction (15) and deposit on the surface, contributing to passive film formation. However, the polarization results indicate that the corrosion product layers composed of Al(OH)_3_, Fe(OH)_2_, and Fe(OH)_3_ are insufficient to form a stable and compact film, leading to a greater i_corr_ of 4.4 μA/cm^2^ without the passivation region.

As seen in Figure 5, the addition of Cr results in a pronounced passivation behavior, with an E_corr_ of 0.055 V, as summarized in Table 8. At this potential, Fe(OH)_2_ is oxidized to Fe(OH)_3_, as listed in Reaction (20). In addition to Fe and Al hydroxides, Cr(OH)_3_ is generated once the potential exceeds –1.058 V, as indicated in Table 3, Reaction (16). Consequently, the extremely low K_sp_ of Cr(OH)_3_ (7 × 10^−31^), Al(OH)_3_ (3 × 10^−34^), and Fe(OH)_3_ (6 × 10^−38^) enables the Fe_60_Al_20_Cr_20_ alloy to form a stable passive film at or below E_corr_ during polarization in 3.5 wt.% NaCl solution. As shown in Table 8, the passive film remains stable up to 0.25 V, beyond which it collapses and the current density rises sharply. These results demonstrate that Cr addition improves resistance to chloride attack at lower potentials; however, at higher potentials, the passive film becomes unstable, leading to severe pitting corrosion.

As illustrated in Figure 5, the simultaneous addition of Mo with Cr further enhances the protective ability of the passive film and increases the pitting potential. According to Table 8, the i_pass_ of Fe_57.7_Al_20_Cr_20_Mo_2.3_ and Fe_55.3_Al_20_Cr_20_Mo_4.7_ is increased from 0.25 μA/cm^2^ to 0.33 μA/cm^2^ and 0.37 μA/cm^2^, while the E_pit_ is elevated from 265 mV to 587 mV and 662 mV (Ag/AgCl), respectively. This improvement of the enlarged passivation region is attributed to the role of Mo during polarization, as described in the previous report that Mo is sacrificed for Cr to keep the original passivation film from the attack of Cl [19]. On the other side, Mo is oxidized to MoO_4_^2−^ at potentials above –0.516 V in Reaction (18). Previous studies have shown that MoO_4_^2−^ ions can compete with Cl^−^ ions for adsorption sites, thereby hindering chloride penetration. Furthermore, in neutral environments, MoO_4_^2−^ may be reduced to Mo(IV), forming MoO_2_ along with other oxides/hydroxides that deposit on the surface and reinforce the passive film [31]. At potentials above –0.006 V, MoO_2_ is oxidized to MoO_3_ in Reaction (21), which further stabilizes the passive film. Consequently, the polarization curves in Figure 5 demonstrate that Mo addition broadens the passivation region and enhances corrosion resistance at higher potentials, with both the passive range and pitting potential significantly increasing as the Mo content rises, as confirmed in Table 8.

#### 3.3.2. Surface Morphologies Before and After Potentiodynamic Polarization Tests in Aerated 3.5 wt.% NaCl Solution

**Figure 6:**
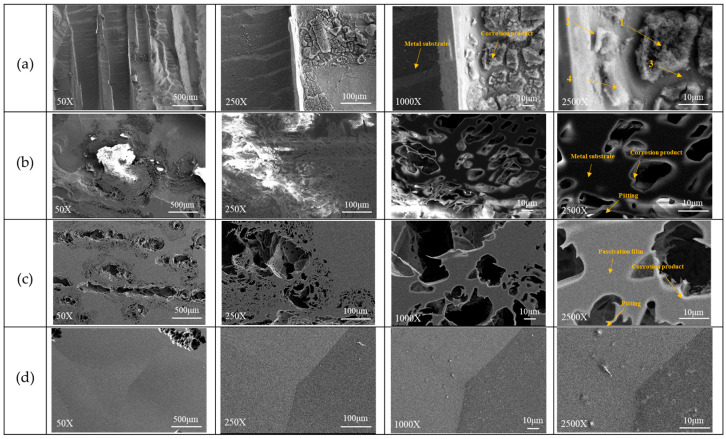
SEI images of (**a**) Fe_80_Al_20_, (**b**) Fe_60_Al_20_Cr_20_, (**c**) Fe_57.7_Al_20_Cr_20_Mo_2.3_, and (**d**) Fe_55.3_Al_20_Cr_20_Mo_4.7_ alloys after potentiodynamic polarization tests in 3.5 wt.% NaCl aerated aqueous solution. The accelerating voltage was 20 kV.

As shown in Figure 6a, severe corrosion of the Fe_80_Al_20_ alloy occurred after potentiodynamic polarization testing in aerated 3.5 wt.% NaCl solution, to the extent that the original grains and grain boundaries became indistinguishable. The polarization curve of Fe_80_Al_20_ presented in Figure 5 indicates that the alloy is unable to form an effective passive film during the anodic process to withstand Cl^−^ attack. As a result, extensive surface corrosion and continuous exposure of the metallic substrate take place, with the severity of corrosion increasing progressively as the potential rises. According to the analysis at point 1 in Table 9, a relatively thick accumulation of corrosion products is observed during the corrosion process, accompanied by high contents of O and Cl. Although Fe(OH)_3_ has a lower solubility product constant (K_sp_) than Al(OH)_3_, Fe(OH)_3_ could be more susceptible to attack by Cl^−^ in chloride-containing environments. Consequently, the Al content in region 1 is higher than that of Fe, and the corrosion products are predominantly composed of deposited Al(OH)_3_. In contrast, in regions 2, 3, and 4, only a thinner corrosion product layer is observed, with significantly lower O content. As a result, signals from the metallic substrate become more prominent, leading to a higher detected Fe signal.

For the Fe_60_Al_20_Cr_20_ alloy (Figure 6b), severe pitting corrosion also occurred after testing in 3.5 wt.% NaCl solution. The grain boundaries present before testing could not be distinguished, and abundant corrosion products were deposited on the underlying metallic substrate, although some remnants of the original alloy surface were still retained. During polarization, OH^−^ in Al(OH)_3_, Cr(OH)_3_, and Fe(OH)_3_ within the passive film was progressively displaced by Cl^−^, leading to continuous release of metal ions and initiation of pitting corrosion. With increasing potential, the corrosion intensified, causing the pits to expand and propagate into the substrate. The released metal ions combined with O^2−^ (OH^−^) and chloride to form oxides/hydroxides, which accumulated on the surface, leaving behind numerous pits of varying sizes.

With the addition of 2.3 at.% Mo, Figure 6c shows that pitting corrosion still occurred; however, more of the original alloy surface was preserved compared to the Cr^−^ free alloys. At lower Mo contents, the passive film was insufficient to completely suppress Cl^−^ penetration, resulting in widespread localized attack. In contrast, when the Mo content was further increased to 4.7 at.%, only a limited amount of corrosion products appeared on the surface, and no extensive corrosion or pitting was observed, as shown in Figure 6d. The specimen surface still displayed clearly visible grains and grain boundaries. Therefore, compared to Fe_80_Al_20_, Fe_60_Al_20_Cr_20_ and Fe_57.7_Al_20_Cr_20_Mo_2.3_, the higher Mo-containing alloy, such as Fe_55.3_Al_20_Cr_20_Mo_4.7_, exhibited improved surface corrosion resistance, with the protective effect becoming more pronounced as the Mo content increased.

#### 3.3.3. Ion Release of the Alloys During Potentiodynamic Tests

According to the results presented in Table 10, no release of metal ions was detected at the corrosion potential of the Fe_80_Al_20_ alloy. This behavior can be attributed to the formation of Fe(OH)_3_ from Fe^2+^ at low potentials, as described by Reaction (15), together with the conversion of Al into Al(OH)_3_ and Al_2_O_3_ via Reactions (13) and (14). Owing to the relatively low solubility product constants (K_sp_) of Fe(OH)_3_ and Al(OH)_3_, these oxides and hydroxides are able to form a protective film on the alloy surface, thereby effectively suppressing metal dissolution. However, when the potential is increased to 0.2 V, the pre-existing protective film breaks down, resulting in severe surface corrosion. Consequently, significant release of metal ions is observed beyond this potential, with the concentrations of Al and Fe ions reaching 7.7 × 10^3^ ppt and 1.6 × 10^4^ ppt, respectively. This behavior is consistent with the polarization curve shown in Figure 5, where the current density increases sharply once the potential exceeds the corrosion potential, indicating a transition of the alloy surface from a passive state to an active dissolution regime accompanied by pronounced corrosion reactions. These results further demonstrate that the protective film is completely destroyed by Cl^−^ attack, which is in good agreement with the presence of Cl-containing corrosion products revealed by the EDS analysis in Table 9. In addition, since Fe is more susceptible to attack in Cl-containing environments, its ion release concentration is comparatively higher.

According to the results presented in Table 11 for specimen Fe_60_Al_20_Cr_20_, only a small amount of Cr ion release was detected near the corrosion potential of the Cr-containing alloy, with a concentration of 9.1 × 10^1^ ppt. This behavior can be attributed to the relatively higher K_sp_ of Cr(OH)_3_ compared with those of Fe(OH)_3_ and Al(OH)_3_, as well as the higher susceptibility of Cr to preferential attack by Cl^−^ in Cl-containing environments, which results in a limited dissolution of Cr ions even at low potentials. In addition, the onset potential for metal ion release shifts to values higher than 0.2 V, which is consistent with the trend observed in Figure 5, where the passive region of the alloy remains stable up to approximately 0.25 V without evident signs of corrosion. These results confirm that the addition of Cr enhances the corrosion resistance of Fe-Al alloys in chloride-containing environments by promoting the formation of protective Cr-, Fe-, and Al-based oxide/hydroxide passive films. Nevertheless, as the potential is further increased, preferential attack on Cr by Cl^−^ leads to partial breakdown of the passive film, allowing Fe and Al to dissolve and be released into the solution. Even so, at 0.2 V, the metal ion release from the Cr-containing alloy remains significantly lower than that from the Cr-free Fe-Al alloy, with only Cr ions being detected, further demonstrating the markedly improved overall corrosion resistance of the alloy.

For the Fe_57.7_Al_20_Cr_20_Mo_2.3_ and Fe_55.3_Al_20_Cr_20_Mo_4.7_ alloys, within the potential range from 0.2 to 0.6 V, the concentrations of released Mo ions are significantly higher than those of Cr ions, as shown in Table 12 and Table 13. This observation indicates that Mo is more susceptible to Cl^−^ attack and undergoes preferential oxidation. During this process, Mo acts as a sacrificial element, thereby protecting Cr, as reported in the previous study [19]. In addition, the released Mo ions can form oxygen-containing anions, which compete with Cl^−^ for adsorption sites on the passive film surface. This competitive adsorption reduces the extent of Cl^−^ adsorption and contributes to the stabilization of the pre-existing passive film. This mechanism accounts for the higher concentration of Mo ions compared with other metal ions. At potentials of 1.0 V or higher, the total ion release from Fe_55.3_Al_20_Cr_20_Mo_4.7_ is on the order of 10^4^, which is lower than that of Fe_57.7_Al_20_Cr_20_Mo_2.3_ (approximately 10^5^). This difference can be attributed to the higher Mo content in the former alloy, which enhances its sacrificial protection capability and more effectively protects the metallic matrix. This interpretation is consistent with the higher pitting potential observed for the Mo-rich alloy.

## 4. Conclusions

With the addition of Cr and Mo to Fe–Al alloys, followed by homogenization heat treatment, the alloys maintained a predominantly body-centered cubic (BCC) structure. The incorporation of Cr alone had a limited effect on hardness, whereas Mo addition significantly enhanced hardness, which can be attributed to indicating that the solid-solution strengthening induced by Mo is more efficient than that by Cr.

The corrosion test results in 0.5 M H_2_SO_4_ solution revealed that the Fe-Al alloy exhibited a relatively high i_crit_ 70,300 μA/cm^2^. With the addition of Cr for specimen Fe_60_Al_20_Cr_20_, its i_crit_ significantly decreased to 12,080 μA/cm^2^ and rapidly passivated, due to not only the formation of Cr(OH)_3_ at a lower potential than the formation of Fe(OH)_2_ but also the lower K_sp_ of Cr(OH)_3_ than that of Fe(OH)_2_, making the anodic and cathodic polarization curves intersected three times and leading to three corrosion potentials. Upon further addition of Mo for Fe_57.7_Al_20_Cr_20_Mo_2.3_ with i_crit_ 78.8 μA/cm^2^, which further decreased with increasing Mo content and was finally much lower than the cathodic polarization current density at lower potential for Fe_55.3_Al_20_Cr_20_Mo_4.7_, resulting in a single intersection at higher potential. This indicates that the further incorporation of Mo easily and effectively stabilizes the formation of the passive film, thereby enhancing the overall corrosion resistance of the alloy in acidic environments.

During potentiodynamic polarization tests in aerated 3.5 wt.% NaCl solution, the Fe_80_Al_20_ alloy continuously undergoes oxidation throughout the polarization process without any passivation region. However, since the solubility products (K_sp_) of the resulting oxides are extremely low, no detectable release of metal ions occurs at the corrosion potential in the absence of Cl^−^ ingress. Upon the addition of Cr, a passive region is established on Fe_60_Al_20_Cr_20_ compared with Fe_80_Al_20_, although the E_pit_ = 265.2 mV remains relatively low. With the incorporation of Mo, both Fe_57.7_Al_20_Cr_20_Mo_2.3_ (E_pit_ = 587.3 mV) and Fe_55.3_Al_20_Cr_20_Mo_4.7_ (E_pit_ = 662.4mV) alloys exhibit a remarkable improvement in raising the pitting potential, which is elevated much higher for the latter alloy due to the increase in Mo content. ICP-MS analyses at the passivation region show that the concentration of Mo is more than that of Cr, especially for Fe_55.3_Al_20_Cr_20_Mo_4.7_, meaning that Mo with enough content is susceptible to Cl^−^ attack and undergoes preferential oxidation to form MoO_4_^2−^, acting as a sacrificial role to protect Cr. Besides, the formation of MoO_4_^2−^ can compete with Cl^−^ ions for adsorption sites on the passive film surface, finally leading to the higher pitting potential.

## Figures and Tables

**Table 1:** Compositions of the samples (at.%).

Alloys	Fe	Al	Cr	Mo
Fe_80_Al_20_	80	20	-	-
Fe_60_Al_20_Cr_20_	60	20	20	-
Fe_57.7_Al_20_Cr_20_Mo_2.3_	57.7	20	20	2.3
Fe_55.3_Al_20_Cr_20_Mo_4.7_	55.3	20	20	4.7

**Table 2:** Vickers hardness test of Fe_80_Al_20_, Fe_60_Al_20_Cr_20_, Fe_57.7_Al_20_Cr_20_Mo_2.3_, and Fe_55.3_Al_20_Cr_20_Mo_4.7_ alloys.

Alloys	Hardness (Hv)
Fe_80_Al_20_	238.4 ± 15
Fe_60_Al_20_Cr_20_	238.6 ± 10
Fe_57.7_Al_20_Cr_20_Mo_2.3_	288.2 ± 22
Fe_55.3_Al_20_Cr_20_Mo_4.7_	298.6 ± 18

**Table 3:** Redox equilibrium potentials of Cr, Fe, Al, and Mo oxides or hydroxides in oxygenated 0.5 M H_2_SO_4_ solution (pH = 0.47).

Electrochemical Reaction	E^0^ (SHE)	0.5 M H_2_SO_4_ (pH = 0.47)
Al(OH)_3_ + 3e^−^ = Al + 3OH^−^	−2.31 V	−1.707 V *(Ag/AgCl)*… (3)
Al_2_O_3_ + 6H^+^ + 6e^−^ = 2Al +3H_2_O	−1.35 V	−1.377 V *(Ag/AgCl)*… (4)
Cr(OH)_3_ + 3e^−^ = Cr + 3OH^−^	−1.3 V	−0.697 V *(Ag/AgCl)*… (5)
MoO_2_ + 4H^+^ + 4e^−^ = Mo + 4H_2_O	−0.152 V	−0.377 V *(Ag/AgCl)*… (6)
Fe(OH)_3_ + 3H^+^ + e^−^ = Fe^2+^ + 3H_2_O	0.074 V	−0.206 V *(Ag/AgCl)*… (7)
Fe(OH)_2_ + 2e^−^ = Fe + 2OH^−^	−0.667 V	−0.064 V *(Ag/AgCl)*… (8)
MoO_4_^2−^ + 8H^+^ + 6e^−^ = Mo + 4H_2_O	0.20 V	−0.034 V *(Ag/AgCl)*… (9)
Fe(OH)_3_ + e^−^ = Fe(OH)_2_ + OH^−^	−0.56 V	0.043 V *(Ag/AgCl)*…(10)
MoO_3_ + 2H^+^ + 2e^−^ = MoO_2_ +H_2_O	0.58 V	0.355 V *(Ag/AgCl)*… (11)
CrO_4_^2−^ + 4H_2_O + 3e^−^ = Cr(OH)_3_ + 5OH^−^	−0.13 V	1.006 V *(Ag/AgCl)*… (12)

**Table 4:** Constant of solubility product (K_sp_) of the related compounds.

Compound	K_sp_
Al(OH)_3_	3 × 10^−34^
Cr(OH)_3_	7 × 10^−31^
Fe(OH)_2_	2 × 10^−15^
Fe(OH)_3_	6 × 10^−38^

**Table 5:** Corrosion characteristics derived from polarization curves in Figure 2.

	Fe_80_Al_20_		Fe_60_Al_20_Cr_20_			Fe_57.7_Al_20_Cr_20_Mo_2.3_			Fe_55.3_Al_20_Cr_20_Mo_4.7_
E_open_ (mV)	−464		165			173			155
E_corr_ (mV)	−435.5 ± 6		−499	−287	59	−388	−323	0.39	29.9 ± 3
i_corr_ (μA/cm^2^)	1473.3 ± 109		3010	11	0.45	26.9	3.02	0.16	0.22 ± 0.05
E_trans_ (mV)	-		981.5			967.2			963.8 ± 10
i_pass_ (μA/cm^2^)	64,294 ± 1518	14.5 ± 0.6	2.43			0.88			2.39 ± 0.2
i_crit_ (μA/cm^2^)	70,300 ± 914		12,080			78.8			-

**Table 6:** EDS analysis results of the Fe_60_Cr_20_Al_20_ alloy shown in Figure 4b_2_.

Atomic%	Al	Fe	Cr	N	O	S
Point 1	18.19	37.83	13.45	-	27.21	3.32
Point 2	21.07	36.19	15.76	19.88	6.07	1.03
Point 3	21.64	57.92	20.44	-	-	-

**Table 7:** Redox equilibrium potentials of Cr, Fe, Al, and Mo oxides or hydroxides in oxygenated 3.5 wt.% NaCl solution (pH = 6.58).

Electrochemical Reaction	E^0^ (SHE)	3.5 wt.% NaCl (pH = 6.58)
Al_2_O_3_ + 6H^+^ + 6e^−^ = 2Al +3H_2_O	−1.35 V	−2.248 V *(Ag/AgCl*)…(13)
Al(OH)_3_ + 3e^−^ = Al + 3OH^−^	−2.31 V	−2.068 V *(Ag/AgCl)*…(14)
Fe(OH)_3_ + 3H^+^ + e^−^ = Fe^2+^ + 3H_2_O	0.074 V	−1.29 V *(Ag/AgCl)*…(15)
Cr(OH)_3_ + 3e^−^ = Cr + 3OH^−^	−1.3 V	−1.058 V *(Ag/AgCl)*…(16)
MoO_2_ + 4H^+^ + 4e^−^ = Mo + 4H_2_O	−0.152 V	−0.738 V *(Ag/AgCl)*…(17)
MoO_4_^2−^ + 8H^+^ + 6e^−^ = Mo + 4H_2_O	0.20 V	−0.516 V *(Ag/AgCl)*…(18)
Fe(OH)_2_ + 2e^−^ = Fe + 2OH^−^	−0.667 V	−0.425 V *(Ag/AgCl)*…(19)
Fe(OH)_3_ + e^−^ = Fe(OH)_2_ + OH^−^	−0.56 V	−0.319 V *(Ag/AgCl)*…(20)
MoO_3_ + 2H^+^ + 2e^−^ = MoO_2_ +H_2_O	0.58 V	−0.006 V *(Ag/AgCl)*…(21)
CrO_4_^2−^ + 4H_2_O + 3e^−^ = Cr(OH)_3_ + 5OH^−^	−0.13 V	0.404 V *(Ag/AgCl)*…(22)

**Table 8:** Corrosion characteristics derived from polarization curves in Figure 3.

	Fe_80_Al_20_	Fe_60_Al_20_Cr_20_	Fe_57.7_Al_20_Cr_20_Mo_2.3_	Fe_55.3_Al_20_Cr_20_Mo_4.7_
E_open_ (mV)	−120	−30	−50	−35
E_corr_ (mV)	−356.3 ± 4	−54.7 ± 0.6	−48.2 ± 0.04	−61.2 ± 0.02
i_corr_ (μA/cm^2^)	4.4 ± 0.4	0.07 ± 0.02	0.08 ± 0.002	0.09 ± 0.02
i_pass_ (μA/cm^2^)	-	0.25 ± 0.02	0.33 ± 0.05	0.37 ± 0.03
E_pit_ (mV)	-	265.2 ± 3	587.3 ± 5	662.4 ± 3

**Table 9:** EDS analysis results of the Fe_80_Al_20_ alloy shown in Figure 6a.

Atomic%	Al	Fe	Cl	Na	O
Point 1	19.35	4.98	9.32	6.11	60.24
Point 2	16.04	43.31	3.10	7.54	30.01
Point 3	17.48	50.35	1.91	4.05	26.21
Point 4	17.63	56.23	1.46	4.63	20.06

**Table 10:** The dissolution ion concentrations of the Fe_80_Al_20_ specimens at the different potentials in 3.5 wt.% NaCl solution.

Metal Ion Concentration (ppt)		
Fe_80_Al_20_	Al	Fe
E_oc_	-	-
0.2 V	7.7 × 10^3^	1.6 × 10^4^
0.6 V	3.2 × 10^5^	2.2 × 10^6^
0.861 V	6.3 × 10^5^	4.1 × 10^6^
1.0 V	6.8 × 10^5^	5.0 × 10^6^
1.2 V	7.0 × 10^5^	6.4 × 10^6^

**Table 11:** The dissolution ion concentrations of the Fe_60_Al_20_Cr_20_ specimens at the different potentials in 3.5 wt.% NaCl solution.

Metal Ion Concentration (ppt)			
Fe_60_Al_20_Cr_20_	Al	Cr	Fe
E_oc_	-	9.1 × 10^1^	-
0.2 V	-	8.3 × 10^1^	-
0.6 V	6.8 × 10^3^	4.7 × 10^3^	4.4 × 10^3^
0.861 V	4.9 × 10^5^	5.9 × 10^5^	2.4 × 10^6^
1.0 V	1.3 × 10^6^	1.4 × 10^6^	4.9 × 10^6^
1.2 V	1.6 × 10^6^	1.1 × 10^6^	4.7 × 10^6^

**Table 12:** The dissolution ion concentrations of the Fe_57.7_Al_20_Cr_20_Mo_2.3_ specimens at the different potentials in 3.5 wt.% NaCl solution.

Metal Ion Concentration (ppt)				
Fe_57.7_Al_20_Cr_20_Mo_2.3_	Al	Cr	Fe	Mo
E_oc_	-	1.0 × 10^2^	-	1.2 × 10^2^
0.2 V	-	1.0 × 10^2^	-	1.2 × 10^2^
0.6 V	-	1.8 × 10^2^	7.3	1.5 × 10^2^
0.861 V	1.3 × 10^3^	3.0 × 10^3^	4.5 × 10^3^	9.0 × 10^2^
1.0 V	1.6 × 10^5^	1.8 × 10^5^	7.5 × 10^5^	6.1 × 10^3^
1.2 V	1.9 × 10^5^	4.5 × 10^5^	1.8 × 10^6^	1.9 × 10^4^

**Table 13:** The dissolution ion concentrations of the Fe_55.3_Al_20_Cr_20_Mo_4.7_ specimens at the different potentials in 3.5 wt.% NaCl solution.

Metal Ion Concentration (ppt)				
Fe_55.3_Al_20_Cr_20_Mo_4.7_	Al	Cr	Fe	Mo
E_oc_	-	8.4	-	-
0.2 V	6.6 × 10^1^	1.5 × 10^2^	-	3.8 × 10^2^
0.6 V	-	1.6 × 10^2^	-	4.0 × 10^2^
0.861 V	8.1 × 10^3^	5.3 × 10^2^	7.8 × 10^2^	2.1 × 10^4^
1.0 V	2.0 × 10^4^	1.0 × 10^4^	2.9 × 10^4^	1.4 × 10^4^
1.2 V	6.4 × 10^4^	6.9 × 10^4^	2.9 × 10^5^	2.6 × 10^5^

## Data Availability

The original contributions presented in this study are included in the article. Further inquiries can be directed to the corresponding author.
